# The Impact of Yoga as an Adjunct to Standard Care on Glycemic Control, Insulin Resistance, Oxidative Stress, and Quality of Life in Individuals with or at Risk of Type 2 Diabetes Mellitus: A Systematic Review and Meta-Analysis

**DOI:** 10.2174/0115733998383494250827215758

**Published:** 2025-09-23

**Authors:** Danish Javed, Madhu Bhargavi Chandra, Varun Malhotra, Sofia Mudda, Padmavathi R, Vishnu Vardhan Karrothu, Madhu Babu Adusumilli, Santenna Chenchula

**Affiliations:** 1 All India Institute of Medical Sciences, Bhopal, M.P, 462020, India;; 2 Wellstar Spalding Regional Hospital, Griffin, Georgia, 30224, USA;; 3 MediCiti Institute of Medical Sciences, Hyderabad, Telangana, 501401, India;; 4 HCA Ocala, Florida, 34471, USA

**Keywords:** Type 2 diabetes mellitus, yoga, glycemic control, insulin resistance, oxidative stress, chronic hyperglycemia

## Abstract

**Introduction:**

Type 2 Diabetes Mellitus (T2DM) is marked by insulin resistance and chronic hyperglycemia. Yoga, a complementary therapy, may improve metabolic outcomes when used with standard care. This systematic review and meta-analysis aimed to evaluate the effects of yoga on glycemic control, insulin resistance, oxidative stress, and psychological well-being in individuals with or at risk of T2DM.

**Methods:**

We conducted a systematic search in PubMed, CAM-QUEST^®^, and the Cochrane Central Register following PRISMA guidelines. We included randomized controlled trials (RCTs) assessing yoga interventions (asanas, pranayama, kriyas, and meditation) alongside standard care. Primary outcomes were fasting blood glucose (FBS), postprandial blood glucose (PPBS), HbA1c, serum insulin, and HOMA-IR. Secondary outcomes included oxidative stress markers (glutathione, malondialdehyde [MDA], superoxide dismutase [SOD]) and psychological outcomes. Meta-analyses were conducted using random-effects models in RevMan 5.4.1.

**Results:**

Fourteen RCTs (n = 1,629 participants) were included. Yoga combined with standard care significantly improved FBS (SMD = -1.60; 95% CI: -2.27 to -0.92), PPBS (SMD = -2.16; *p* = 0.03), HbA1c (SMD = -0.92; 95% CI: -1.59 to -0.26), and HOMA-IR (SMD = -1.28; *p* = 0.002). MDA levels were significantly reduced, though glutathione and SOD showed no significant changes. Psychological well-being improved in several trials.

**Discussion:**

Yoga appears effective in improving glycemic outcomes and insulin resistance in patients with or at risk of T2DM. It may also reduce oxidative stress and enhance psychological well-being, highlighting its potential as a holistic intervention.

**Conclusion:**

Yoga, when integrated with standard care, offers clinically relevant benefits in managing T2DM and related metabolic risks. Further high-quality, multi-centered trials using standardized protocols are needed to validate and generalize these findings.

## 1 INTRODUCTION

Diabetes Mellitus is a chronic metabolic disorder marked by elevated blood glucose levels and is a major global health priority, ranked as the eighth leading cause of death and disability [1]. Diabetes has become an international health emergency, with over 10% of the world’s population affected in 2021, a figure expected to rise by 46% by 2045 [1]. Uncontrolled diabetes leads to serious complications, both microvascular and macrovascular, imposing significant healthcare burdens [2]. Type 2 Diabetes Mellitus (T2DM), accounting for 90% of cases, is characterized by insulin resistance (IR) and inadequate insulin secretion [3]. IR impairs the body's ability to regulate glucose, increasing production in the liver while reducing glucose uptake in tissues. This, combined with beta-cell dysfunction, exacerbates hyperglycemia, advancing T2DM [3]. Obesity and sedentary lifestyles are major risk factors, but they can be addressed, reducing the risk of IR [3]. Medications remain the primary approach for the treatment of T2DM. However, preliminary studies have shown that practicing yoga is effective in controlling blood sugar levels in persons with T2DM [4].

With roots over 3,000 years old, yoga seeks to balance body, mind, and emotions. It is the most common complementary or alternative medicine (CAM) therapy, and the therapeutic benefits of yoga have only recently gained attention in modern healthcare [5]. Studies have shown that 3-20% of the population uses CAM therapy for the treatment of T2DM [6, 7]. Yoga has positively affected various health outcomes, including diabetes prevention, weight management, blood pressure regulation, and lipid profile improvement [6, 7]. Additionally, yoga may help prevent cardiovascular events and manage diabetes-related complications, integrating ancient wisdom with modern healthcare practices [8]. Research has explored the effects of yoga across biochemical, genetic, neuromuscular, and other health domains, with compelling evidence that it addresses key mechanisms involved in diabetes management. According to the American Diabetes Association, behavioral management is crucial for achieving diabetes treatment goals, and yoga can play a transformative role by promoting discipline in diet and exercise, where many patients struggle [9].

The objective of this systematic review and meta-analysis is to evaluate the effects of yoga, when used as an adjunct to standard care, on glycemic control, insulin resistance, oxidative stress markers, and psychological well-being in individuals with or at risk of Type 2 Diabetes Mellitus (T2DM), including those with pre-diabetes. The primary outcomes assessed include changes in fasting blood glucose, postprandial blood glucose, HbA1c, serum insulin levels, and Homeostasis Model Assessment of Insulin Resistance (HOMA-IR). Secondary outcomes encompass oxidative stress markers—such as glutathione, malondialdehyde (MDA), and superoxide dismutase (SOD)—psychological well-being (assessed through depression, anxiety, and stress scales), the Indian Diabetes Risk Score (IDRS), quality of life (QoL), and HOMA-IR.

## 2 METHODS

This systematic review and meta-analysis adhered to the Preferred Reporting Items for Systematic Reviews and Meta-Analyses (PRISMA) guidelines and was registered with the International Prospective Register of Systematic Reviews (PROSPERO; CRD42022310593) [10].

### 2.1 Search Strategy

A comprehensive search was conducted across PubMed/MEDLINE, CAM-QUEST^®^, and the Cochrane Central Register of Controlled Trials (CENTRAL) from database inception to October 2024. Grey literature and reference lists of relevant studies were also screened. The search strategy combined Medical Subject Headings (MeSH) and free-text terms related to yoga and Type 2 Diabetes Mellitus using: (“yoga” or “asanas” or “pranayama” or “meditation”) AND (“Diabetes Mellitus, Type 2” OR “Type 2 Diabetes Mellitus” OR “T2DM” OR “Glycemic Control” OR “HbA1c”) AND (“Randomized Controlled Trial” OR “RCT”).

Search filters were applied to include only articles published in English. No restrictions were placed on publication year.

### 2.2 Eligibility Criteria

This systematic review included studies involving participants diagnosed with Type 2 Diabetes Mellitus or pre-diabetes. The interventions must include yoga-based practices such as kriyas, asanas, pranayama, and meditation in conjunction with standard care. Comparators must involve standard care, with or without a placebo or conventional exercise. The studies must assess outcomes like fasting blood glucose, HbA1C, BMI, insulin resistance, and blood pressure, and only randomized controlled trials (RCTs) are eligible. Excluded studies include quasi-randomized trials, observational studies, cohort studies, case reports, systematic reviews, and those involving severe or critical cases.

### 2.3 Intervention Characteristics

Yoga interventions varied across studies but typically included structured sessions ranging from 30 to 90 minutes per session, 3 to 7 times per week, for durations ranging from 4 weeks to 6 months. Intensity levels were primarily moderate and supervised by certified instructors. Most interventions combined multiple components of yoga (*e.g.*, asanas, pranayama, and meditation), though some focused on specific practices.

### 2.4 Study Selection

Two reviewers independently screened the titles and abstracts of all retrieved citations. Full texts of potentially eligible studies were assessed for inclusion. Disagreements were resolved through discussion or arbitration by a third reviewer. EndNote and Rayyan tools were used for reference management and blinded screening.

### 2.5 Data Extraction

Data from the included RCTs were systematically recorded in a standardized predesigned Excel sheet. Extracted data included participant characteristics, study design, interventions, comparators, outcomes, and key results.

### 2.6 Risk of Bias Assessment

Two reviewers independently assessed the risk of bias using the Cochrane Risk of Bias 2.0 tool across five domains: randomization process, deviations from intended interventions, missing outcome data, measurement of outcomes, and selection of the reported result. Judgments were categorized as “low risk,” “some concerns,” or “high risk.” Disagreements were resolved by consensus.

### 2.7 Missing Data

Efforts were made to contact authors for missing or unclear data. Attrition rates, methods for handling missing data (*e.g.*, last observation carried forward), and any imputation techniques were recorded. Sensitivity analyses were performed where applicable.

### 2.8 Assessment of Heterogeneity

Heterogeneity across studies was assessed using the Chi-square (χ^2^) test and the I^2^ statistic, with I^2^ > 75% indicating substantial heterogeneity. Clinical and methodological heterogeneity were also considered.

### 2.9 Data Synthesis and Statistical Analysis

Pooled effects were calculated using random-effects models to account for between-study variability. Standardized mean differences (SMDs) or mean differences (MDs) with 95% confidence intervals (CIs) were reported. Analyses were performed using Review Manager (RevMan) version 5.4.1. Sensitivity analyses were conducted to evaluate the robustness of findings. Publication bias was assessed using Egger’s linear regression test, a standard method to evaluate potential asymmetry in the distribution of study effect sizes, which may indicate selective publication of results. Where data allowed, subgroup analyses were planned based on intervention duration, yoga type, and participant characteristics.

### 2.10 Grading the Quality of Evidence

The certainty of the evidence for each outcome was assessed using the GRADE (Grading of Recommendations Assessment, Development, and Evaluation) approach. This method evaluates evidence across five domains: risk of bias, inconsistency, indirectness, imprecision, and publication bias. Each outcome was rated as having high, moderate, low, or very low certainty, depending on the presence and severity of limitations in these domains.

We applied GRADE to all primary and secondary outcomes assessed in the meta-analysis. The GRADEpro software was used to facilitate transparent evaluation and presentation of evidence certainty. Outcomes with ≥10 studies were also considered for publication bias assessment. Downgrading decisions were made based on observed heterogeneity (I^2^ > 75%), sample size limitations, wide confidence intervals, and study design limitations.

## 3 RESULTS

### 3.1 Search Results

A total of 114 records were identified through database searches. After removing 38 duplicates and excluding 41 irrelevant records, 35 citations were screened. Of these, 10 articles were excluded, 3 due to non-availability of full-text articles and 7 based on title and abstract review. A total of 25 papers were assessed for eligibility, with 11 excluded for various reasons, including variation in RCT design (3), inadequate follow-up (2), missing values (3), irrelevant indices (2), and other outcomes (1). Ultimately, 14 studies were included in this meta-analysis, as shown in the PRISMA 2020 flowchart (Fig. **1**).

### 3.2 Characteristics of Included Studies:

A total of 22 studies were included in the systematic literature review; however, for the meta-analysis, only 14 randomized controlled trials, with 1629 patients, were eligible [11-33]. The characteristics of the included studies, detailing interventions such as *kriyas, asanas, pranayama*, and meditation, are summarized in supplementary Table **S1**.

### 3.3 Meta-analysis of Yoga Interventions

The meta-analysis comparing yoga interventions with control groups demonstrated significant improvements in several key clinical parameters favoring the yoga group:

#### 3.3.1 Fasting Blood Sugar (FBS)

A significant reduction was observed with a standardized mean difference (SMD) of -1.60 (95% CI: -2.27 to -0.92; *p* < 0.00001), with high heterogeneity (Chi^2^ = 372.23, I^2^ = 97%) (Fig. **2**).

#### 3.3.2 Postprandial Blood Sugar (PPBS)

A marked decrease was noted with an SMD of -2.16 (95% CI: -4.04 to -0.27; *p* = 0.03; Chi^2^ = 331.37, I^2^ = 98%) (Fig. **3**).

#### 3.3.3 HbA1c

A significant reduction was achieved, with an SMD of -0.92 (95% CI: -1.59 to -0.26; *p* = 0.007; Chi^2^ = 115.59, I^2^ = 96%) (Fig. **4**).

#### 3.3.4 Serum Insulin Levels

A substantial decline was reported, with an SMD of -1.45 (95% CI: -2.73 to -0.18; *p* = 0.03; Chi^2^ = 156.68, I^2^ = 97%) (Fig. **5**).

#### 3.3.5 HOMA-IR (Insulin Resistance)

A significant improvement in insulin sensitivity was noted, with an SMD of -1.28 (95% CI: -2.10 to -0.46; *p* = 0.002; Chi^2^ = 46.16, I^2^ = 94%) (Fig. **6**).

### 3.4 Secondary Outcomes

Secondary outcomes related to oxidative stress markers were also analyzed:

#### 3.4.1 Serum Glutathione Levels

Although the effect was not statistically significant, an SMD of 1.04 was observed (95% CI: -0.60 to 2.67; *p* = 0.21; Chi^2^ = 15.04, I^2^ = 93%) (Fig. **7**).

#### 3.4.2 Malondialdehyde (MDA) Levels

A significant reduction was found, with an SMD of -4.06 (95% CI: -7.30 to -0.82; *p* = 0.01; Chi^2^ = 112.64, I^2^ = 98%) (Fig. **8**).

#### 3.4.3 Superoxide Dismutase (SOD) Levels

No significant effect was observed, with an SMD of -0.08 (95% CI: -3.65 to 3.49; *p* = 0.92; Chi^2^ = 258.25, I^2^ = 99%) (Fig. **9**).

### 3.5 Certainty of Evidence

The GRADE assessment highlighted moderate certainty for FBS and HbA1c, while other outcomes were downgraded to low or very low certainty due to heterogeneity, inconsistency, imprecision, and/or risk of bias. No outcomes were supported by high-certainty evidence.

### 3.6 Risk of Bias across Studies

Overall, the assessment revealed some limitations related to randomization and blinding. However, most studies were deemed to provide sufficiently reliable data for inclusion in the meta-analysis (Fig. **10**).

The results indicate that yoga interventions significantly improve glycemic control, reduce insulin resistance, and lower oxidative stress markers in patients with Type 2 Diabetes Mellitus. Combined with standard care, regular yoga practice is a practical approach for managing diabetes and enhancing overall metabolic health. The risk of bias in the included studies was evaluated using the Cochrane Collaboration’s tool, and the findings are summarized in a risk of bias graph.

### 3.7 Publication Bias

To assess publication bias for the provided meta-analysis on fasting blood sugar (≥10 studies), an Egger’s test was performed using the standardized mean differences (SMDs) and their standard errors (SEs) derived from the 95% confidence intervals. The test revealed no significant asymmetry (intercept = -2.22, SE = 1.34, t = -1.66, *p* > 0.05), indicating no strong evidence of publication bias. Egger’s test regression intercept: -2.22 (SE = 1.34, *p* > 0.05), suggesting no significant publication bias. The funnel plot is likely symmetrical, supporting the absence of bias.

### 3.8 Evidence Quality and Summary of Findings and Recommendations

The certainty of the evidence obtained using the GRADE approach for key glycaemic outcomes such as fasting blood sugar, postprandial blood sugar, and HbA1c ranged from moderate to low, suggesting that yoga combined with standard care is likely to offer meaningful clinical benefits in individuals with or at risk of Type 2 Diabetes Mellitus. The evidence for fasting blood sugar and HbA1c was of moderate certainty, indicating that strong recommendations may be appropriate for integrating yoga into standard diabetes management. However, the evidence for other outcomes such as HOMA-IR, oxidative stress markers (malondialdehyde, glutathione, superoxide dismutase), and serum insulin levels was of low to very low certainty, primarily due to heterogeneity, small sample sizes, and study design limitations. As such, while the overall evidence supports the potential of yoga as an effective adjunct intervention, further high-quality randomized controlled trials are needed to strengthen confidence in these findings and fully establish its role in improving metabolic and oxidative outcomes in this population (Table **S2**).

## 4 DISCUSSION

This systematic review and meta-analysis provides compelling evidence supporting yoga as an effective adjunct therapy for managing Type 2 Diabetes Mellitus (T2DM). The findings demonstrate significant improvements in key glycemic parameters, insulin resistance, and oxidative stress markers, underscoring the therapeutic potential of yoga for individuals with T2DM. Yoga is among various mind-body practices that leverage the interplay between the mind, body, and behavior to enhance overall health while improving physical and mental well-being [34-37].

The results of this study revealed that yoga interventions significantly reduced fasting blood sugar (FBS), postprandial blood sugar (PPBS), and HbA1c levels, indicating improved glycemic control. Notably, a significant reduction in HbA1c was achieved with a standardized mean difference (SMD) of -0.92 (95% CI: -1.59 to -0.26; *p* = 0.007; Chi^2^ = 115.59, I^2^ = 96%). This reduction aligns with previous studies suggesting that yoga can effectively lower HbA1c levels by improving insulin sensitivity and enhancing glucose uptake in peripheral tissues [38]. The observed effect on HbA1c, a key long-term marker for glycemic control, falls within a clinically meaningful range. A 0.5% to 1.0% reduction in HbA1c is generally considered significant and associated with a reduced risk of diabetes-related complications. This suggests that yoga may improve clinical outcomes for individuals with T2DM [39-41].

Additionally, the reductions in FBS and PPBS levels further support the clinical relevance of yoga in managing T2DM, as these changes also reflect improvements in overall glycemic control and a reduced risk of long-term complications. The reduction in FBS (SMD: -1.60, 95% CI: -2.27 to -0.92) and PPBS (SMD: -2.16, 95% CI: -4.04 to -0.27) underscores yoga's potential to lower blood glucose levels by enhancing insulin sensitivity and glucose uptake in peripheral tissues [38]. These changes may be attributed to yoga's physiological and psychological benefits, such as improved autonomic function, reduced cortisol levels, and enhanced beta-cell function [42]. This finding is consistent with other systematic reviews highlighting similar benefits [42-44].

The observed decline in HOMA-IR (SMD: -1.28, 95% CI: -2.10 to -0.46) further reinforces yoga's role in mitigating insulin resistance. This improvement may be due to yoga's ability to reduce oxidative stress, modulate inflammatory pathways, and promote weight loss—all key factors contributing to improved insulin sensitivity [4]. Additionally, the reduction in serum insulin levels corroborates these findings, emphasizing yoga's efficacy in addressing the underlying mechanisms of insulin resistance [45]. Evidence also suggests that yoga may help reduce energy intake and promote physical activity in adults with overweight or obesity, further enhancing metabolic outcomes [46].

This meta-analysis also highlighted significant reductions in oxidative stress markers, particularly malondialdehyde (MDA) levels (SMD: -4.06, 95% CI: -7.30 to -0.82). MDA, a well-established marker of lipid peroxidation, reflects oxidative damage, and its reduction suggests improved antioxidant defenses in individuals practicing yoga. These findings are consistent with prior studies reporting that yoga reduces oxidative stress and enhances antioxidant systems, ultimately contributing to improved metabolic outcomes in T2DM [44, 47, 48]. Although serum glutathione levels showed no statistically significant change, the trend toward improvement aligns with yogas' potential to bolster endogenous antioxidant systems. Similarly, the non-significant effect on superoxide dismutase (SOD) levels may reflect variability in study designs, differences in yoga interventions, or insufficient durations of practice. Future studies employing standardized protocols are warranted to clarify yoga's impact on oxidative stress markers [49].

Beyond physiological outcomes, yoga's holistic approach promotes psychological well-being, which is critical for patients with T2DM. Although this meta-analysis primarily focused on biochemical outcomes, evidence from the included studies suggests that yoga can reduce depression, anxiety, and stress, thereby improving overall quality of life (QoL) [50-53]. Improved psychological health may indirectly influence glycemic control by fostering better adherence to lifestyle modifications and medical regimens [50-53]. Additionally, evidence indicates that yoga significantly improves various cardiometabolic and stress-related risk factors, including lipid profiles, blood pressure, body mass index (BMI), waist-to-hip ratio, and cortisol levels in adults with T2DM [54]. Yoga has also been shown to enhance health-related fitness, particularly muscle strength and cardiorespiratory fitness, among patients with T2DM [54, 55]. A 24-week yoga-based intervention (YBI) significantly improved symptoms of diabetic autonomic neuropathy and reduced stress, outperforming the ADA exercise regimen in enhancing the quality of life (WHO-5 Wellness) and alleviating depression symptoms [56].

Therefore, the findings of this meta-analysis have significant clinical implications [57-59]. Yoga, as a cost-effective and accessible intervention, can complement standard care in T2DM management. Healthcare providers should consider incorporating structured yoga programs into diabetes care plans to enhance glycemic control and overall metabolic health. Future research should focus on addressing the limitations identified in this study. Standardized yoga protocols, longer follow-up durations, and larger sample sizes are needed to confirm the long-term benefits of yoga for T2DM. Exploring the molecular mechanisms underlying yoga’s effects may provide deeper insights into its therapeutic potential.

While yoga demonstrates promise as an adjunct therapy for T2DM, the GRADE assessment underscores the need for higher-quality evidence to solidify recommendations. The moderate certainty for glycemic outcomes supports its provisional use in clinical settings. Still, the inconsistent and low-certainty findings for insulin resistance and oxidative stress markers highlight critical knowledge gaps. Future research must address methodological shortcomings to clarify yoga’s role in holistic diabetes management.

This study’s strength lies in its rigorous methodology, including randomized controlled trials (RCTs) and a comprehensive assessment of glycemic, oxidative, and psychological outcomes. However, several limitations must be acknowledged. High heterogeneity was observed in specific outcomes, such as postprandial blood glucose (PPBS) and HbA1c. This heterogeneity could be due to differences in study designs, participant characteristics, types of yoga interventions, durations of practice, and outcome measurement tools. Second, due to available data constraints and reporting variability, subgroup analyses could not be conducted at this stage. This limited our ability to explore the influence of factors such as duration and frequency of yoga practice, age, baseline glycemic status, and type of yoga components (*e.g.*, pranayama, asanas, meditation) on clinical outcomes. Additionally, many included studies had small sample sizes and a moderate risk of bias, particularly related to randomization and blinding, which may influence the strength and generalizability of the evidence. Furthermore, nearly all included studies were conducted in India, which, while reflecting the current body of research, limits the external validity and generalizability of the findings. Cultural and healthcare system differences may impact the applicability of these results to other populations.

Future studies should aim for more rigorous designs with standardized yoga protocols and longer follow-up durations to evaluate the sustainability of yoga's benefits. Subgroup analyses based on intervention characteristics and participant demographics will also be valuable in identifying which patient populations may benefit the most from yoga interventions in managing T2DM. Expanding research beyond India will enhance the external validity and provide insights into yoga’s global applicability in managing T2DM.

## CONCLUSION

This systematic review and meta-analysis suggests that when integrated with standard care, yoga interventions may improve glycemic control, insulin resistance, and oxidative stress markers in individuals with Type 2 Diabetes Mellitus (T2DM). Yoga appears to be a promising, accessible, and holistic adjunct therapy. While several studies reported enhancements in psychological well-being and quality of life, the evidence for these outcomes remains limited and requires further substantiation. Therefore, additional high-quality, methodologically rigorous studies with standardized intervention protocols and more extended follow-up periods are needed to confirm these findings and support the integration of yoga into routine diabetes care.

## Figures and Tables

**Fig. (1):**
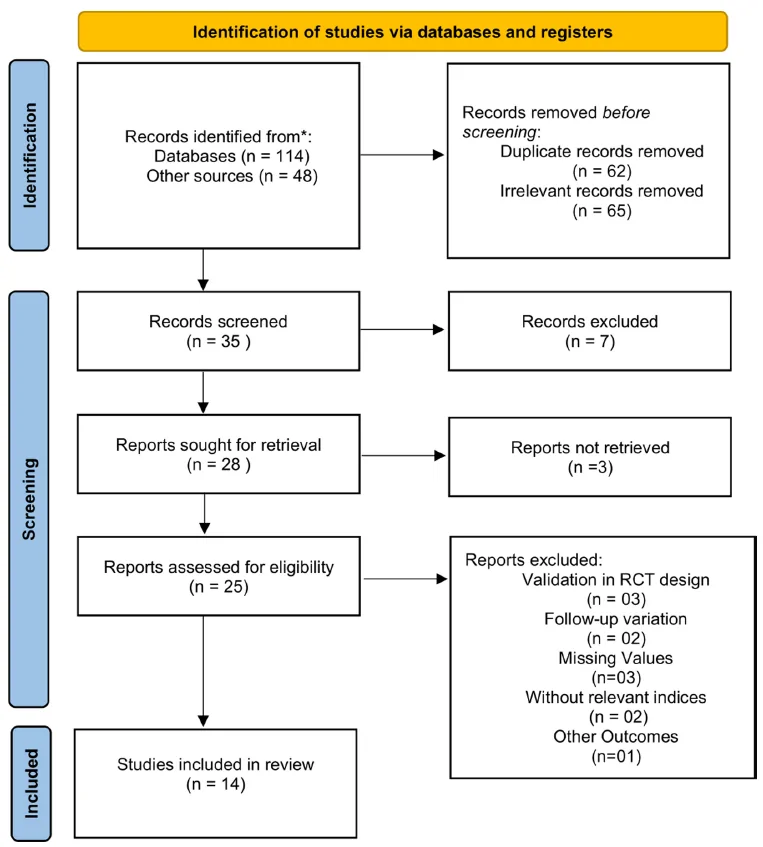
Selection of studies to be included in the systematic review and meta-analysis.

**Fig. (2):**
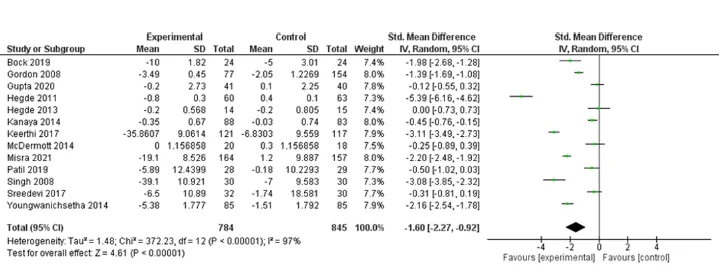
Forest plot of the fasting blood sugar.

**Fig. (3):**
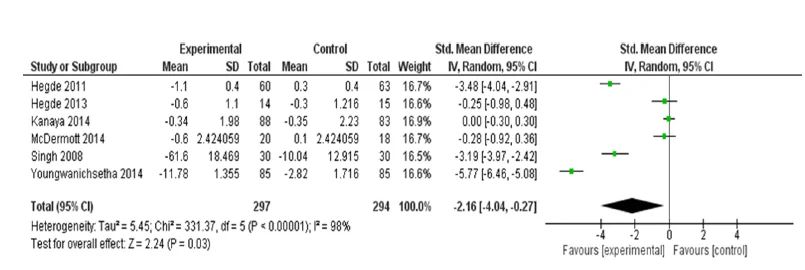
Forest plot of the PPBS.

**Fig. (4):**
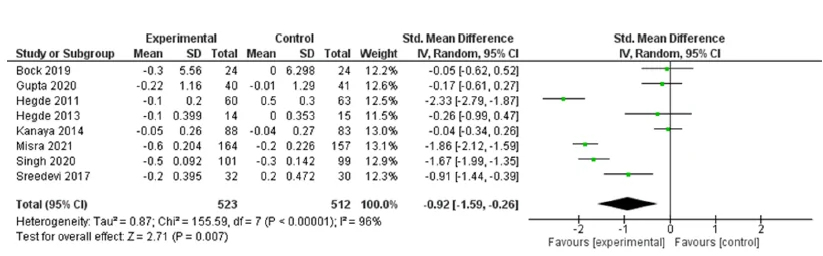
Forest plot of the HbA1C.

**Fig. (5):**
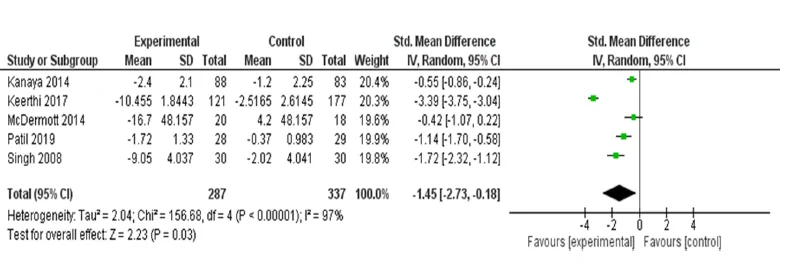
Forest plot of the S. Insulin.

**Fig. (6):**
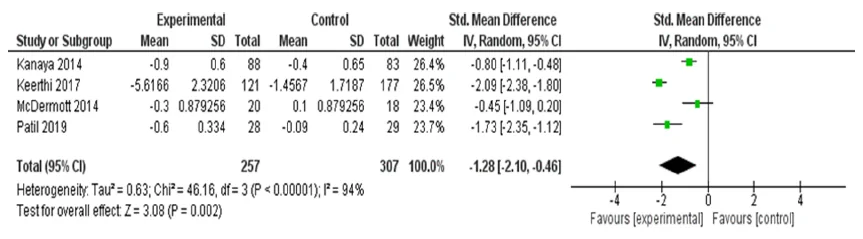
Forest plot of the HOMA-IR.

**Fig. (7):**
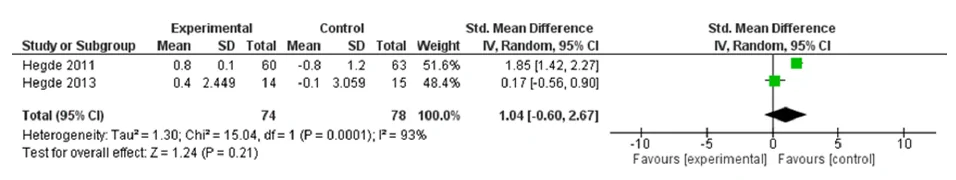
Forest plot of the S.Glutathione.

**Fig. (8):**
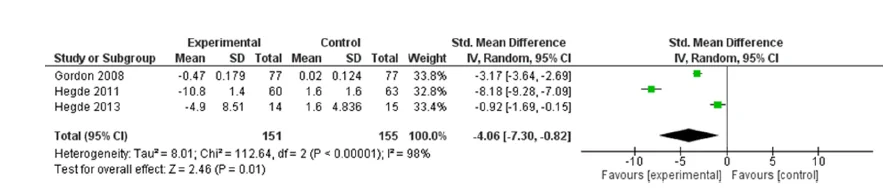
Forest plot of the MDA.

**Fig. (9):**
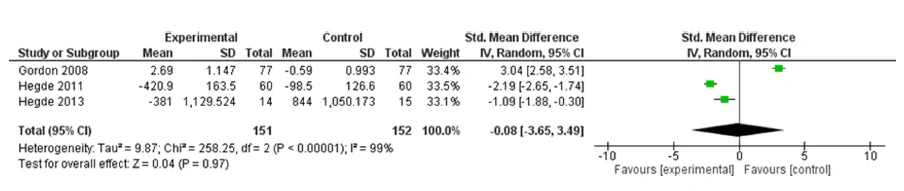
Forest plot of the SOD.

**Fig. (10):**
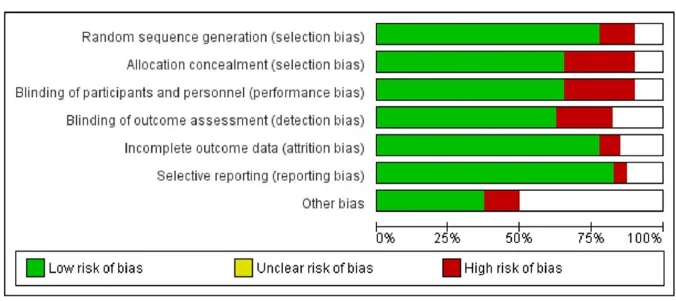
Risk of bias graph.

## Data Availability

Not applicable.

## LIST OF ABBREVIATIONS

ADA: American Diabetes Association
CAM: Complementary and Alternative Medicine
CENTRAL: Cochrane Central Register of Controlled Trials
FBS: Fasting Blood Glucose
HbA1c: Glycated Hemoglobin
HOMA-IR: Homeostasis Model Assessment of Insulin Resistance
IDRS: Indian Diabetes Risk Score
MDA: Malondialdehyde
PPBS: Postprandial Blood Glucose
PROSPERO: International Prospective Register of Systematic Reviews
QoL: Quality of Life
RCT: Randomized Controlled Trial
SMD: Standardized Mean Difference
SOD: Superoxide Dismutase
T2DM: Type 2 Diabetes Mellitus

## STANDARDS OF REPORTING

PRISMA guidelines and methodology were followed.

## SUPPLEMENTARY MATERIAL

PRISMA checklist is available as supplementary material on the publisher’s website along with the published article.
